# Employing the Aviation Model to Reduce Errors in Robotic Gynecological Surgery: A Narrative Review

**DOI:** 10.3390/healthcare12161614

**Published:** 2024-08-13

**Authors:** Stefano Restaino, Federico Paparcura, Martina Arcieri, Giulia Pellecchia, Alice Poli, Valerio Gallotta, Salvatore Gueli Alletti, Stefano Cianci, Vito Andrea Capozzi, Giorgio Bogani, Alessandro Lucidi, Marko Klarić, Lorenza Driul, Vito Chiantera, Fabrizio Dal Moro, Giovanni Scambia, Giuseppe Vizzielli

**Affiliations:** 1 Department of Maternal and Child Health, Obstetrics and Gynecology Clinic, University Hospital of Udine, 33100 Udine, Italy; martina.arcieri@asufc.sanita.fvg.it (M.A.); lorenza.driul@uniud.it (L.D.); giuseppe.vizzielli@uniud.it (G.V.); 2 PhD School in Biomedical Sciences, Gender Medicine, Child and Women Health, University of Sassari, 07100 Sassari, Italy; 3 Department of Medicine (DMED), University of Udine, 33100 Udine, Italy; paparcura.federico@spes.uniud.it (F.P.); pellecchia.giulia001@spes.uniud.it (G.P.); poli.alice@spes.uniud.it (A.P.); 4 Department of Woman, Child Health and Public Health, Fondazione Policlinico Universitario A. Gemelli IRCCS, 00168 Rome, Italy; valerio.gallotta@policlinicogemelli.it (V.G.); giovanni.scambia@policlinicogemelli.it (G.S.); 5 Department of Obstetrics and Gynecology, Ospedale Buccheri La Ferla—Fatebenefratelli, 90123 Palermo, Italy; guelialletti.salvatore@fbfpa.it; 6 Obstetrics and Gynecology Unit, Department of Human Pathology of Adult and Childhood “G. Barresi”, University of Messina, 98125 Messina, Italy; stefano.cianci@unime.it; 7 Department of Medicine and Surgery, University Hospital of Parma, 43126 Parma, Italy; vitoandrea.capozzi@studenti.unipr.it; 8 Gynecologic Oncology Unit, Department Oncologic Surgery, Fondazione IRCCS Istituto Nazionale dei Tumori di Milano, 20133 Milan, Italy; giorgio.bogani@istitutotumori.mi.it; 9 Department of Gynecological, Obstetrical and Urological Sciences, “Sapienza” University of Rome, 00185 Rome, Italy; 10 Department of Obstetrics and Gynecology, University of Chieti, 66100 Chieti, Italy; ginecologia.lucidi@gmail.com; 11 Department of Obstetrics and Gynaecology, Clinical Hospital Center of Rijeka, 51000 Rijeka, Croatia; marko.klaric2@medri.uniri.hr; 12 Department of Health Promotion, Mother and Child Care, Internal Medicine and Medical Specialties (PROMISE), University of Palermo, 90133 Palermo, Italy; vito.chiantera@unipa.it; 13 Unit of Gynecologic Oncology, National Cancer Institute—IRCCS—Fondazione “G. Pascale”, 80138 Naples, Italy; 14 Department of Surgery, Oncology, and Gastroenterology, Urology Clinic, University of Padua, 35124 Padova, Italy; fabrizio.dalmoro@unipd.it; 15 Universita’ Cattolica del Sacro Cuore, 00168 Rome, Italy

**Keywords:** robotic surgery, training, simulation, checklist, aviation

## Abstract

The operating room is the environment where harm to the patient is most likely. Robotic surgery was listed as one of the top 10 health hazards as late as 2020. Taking inspiration from other fields of application, such as aeronautics, checklists have been increasingly implemented in medical practice over the years, becoming essential components of the operating theatre. In addition to checklists, simulation has taken on a fundamental importance in reducing errors. This paper aims to provide a narrative review to assess the importance of checklists and training in robotic surgery and how they improve the outcome. A comprehensive literature search from January 2000 to September 2023 was conducted. A total of 97 articles were included in the initial search. Eleven studies were deemed relevant and were considered eligible for full-text reading. Among these, ten studies focused on the analysis of training effectiveness. An article in our review assessed the benefits of introducing checklists in the operating room. Innovations created in aviation, such as checklists and simulation, have entered the medical field to prevent human error. Developing dedicated checklist and surgical teams, through theoretical and practical training, has become essential in modern medicine. Tools such as checklists, training, and simulation are among the best methods to reduce adverse medical events.

## 1. Introduction

The theory of active and latent failures was introduced by James Reason in the book entitled “Human Error”, taking the name of the “Swiss cheese model” [1]. According to Reason, accidents within complex systems, such as healthcare, are caused by errors on four levels within a system: unsafe acts, preconditions for unsafe acts, supervisory factors, and organizational influences [1,2].

The operating room is a high-risk setting, where patient safety should be prioritized at all stages of the procedure. Indeed, the operating room is the environment where harm to the patient is most likely [3]. During a robot-assisted surgery, the surgical team faces several challenges [4,5]. Healthcare professionals are responsible for ensuring effective communication among themselves while interacting with a complex machine. Some research has demonstrated that adverse events in surgery are mainly due to failures in non-technical skills, such as communication, teamwork, leadership, and decision making [6,7]. Despite significant progress and recent technological advancements, robotic surgery was listed as one of the top 10 health hazards as late as 2020 [8].

As we know, checklists and operator training have a fundamental role in error prevention by improving surgeons’ skills and reducing their errors in the theatre.

Aviation safety checklists can usually be traced back to the 1930s, before the Second World War. The initial flight of the Boeing Model 299, later renamed the B-17 bomber, failed due to human error that resulted in the deaths of both pilots. To reduce the risk of human error, military pilots developed a checklist to assist the operator in the safe control and operation of the aircraft, which later became an integral part of the Allied aviation success during World War II [9]. Since then, pilot checklists have become essential to pre-flight preparations and flight operations. Checklists are memory-supporting tools with predetermined tasks to complete a process [10].

Taking inspiration from other fields of application, such as aeronautics, checklists have been increasingly implemented in medical practice over the years, becoming essential components of the operating theatre.

The importance of checklists is highlighted because all international hospital accreditation systems require creating and implementing checklists as a fundamental requirement in medical care procedures, with a particular emphasis on surgical practices.

Both the Agency for Healthcare Research and Quality and the Institute of Medicine suggest that patient safety can be improved in healthcare by implementing aviation crew resource management (CRM). CRM emphasizes six key areas: fatigue management, team creation and management, red flag recognition, cross-checking and communication, decision making, and performance feedback [11].

In addition to checklists, simulation has taken on a fundamental importance in reducing errors [12].

The history of simulators began in the 1920s, when pilots started using scale aircraft models to evaluate the performance of new designs. Later, in the 1930s, pilots began employing mechanical devices to simulate the sensations of flight. The first real simulator was the Link Trainer. Following the Air Mail scandal, during which the Army Air Corps took control of U.S. Air Mail transportation, the Link Trainer saw its first military sales. In the first few months, twelve pilots lost their lives due to their unfamiliarity with instrument flight conditions. This tragedy prompted the Air Corps to consider Link’s pilot trainer as a potential solution. A significant demonstration of the trainer’s effectiveness occurred in 1934, when Edwin Link successfully flew in adverse weather conditions that the Air Corps evaluation team had deemed impossible [13]. As a result, on 23 June 1934, the Air Corps placed an order for the first six Link Trainers [14,15]. In 1937, American Airlines became the first commercial airline to purchase a Link Trainer [16]. Subsequently, Link Trainers were also sold to the U.S. Navy and to several foreign nations [17]. Nowadays, flight simulators have become an essential part of modern aviation.

Similarly, the ability of simulators to reproduce clinical scenarios has resulted in them being used in obstetric and gynecological education. Since the 1960s, simulations with mannequins have been used in medical training and, over time, have evolved with new technologies such as computer graphics and virtual reality [18].

In recent years, agencies have been created to certify simulation programs. The GESEA Educational Programme (Gynaecological Endoscopic Surgical Education and Assessment), supported by the European Society of Gynaecological Oncology (ESGO) and Society of European Robotic Gynaecological Surgery (SERGS), is the most important in the field of minimally invasive gynecological surgery. GESEA is a structured educational program for gynecological endoscopy. It trains and certifies doctors in surgical knowledge and practical skills related to surgical proficiency, primarily through simulation. It involves training in robotic surgery through a theoretical exam and simulation exercises to improve psychomotor skills when using the robot [19]. This study aimed to perform a narrative review to assess the importance of checklists and training in robotic surgery and to ascertain how they improve outcomes.

## 2. Materials and Methods

A comprehensive literature search from January 2000 to September 2023, in the English language, was conducted. The search terms included a combination of the following items using AND: “robotic training”, “simulation”, “operator training”, “gynecology”, “robotic surgery”, “DaVinci training”, “surgical simulation”, “gyn surgical training”, “checklist”, and “surgical safety checklist”. The terms “robotic surgery” and “gynecology” were included in all the combinations used. The sources considered for inclusion included original articles, literature reviews, and textbook chapters. The articles were subsequently selected based on the evaluation of their abstracts; only full-text articles were considered eligible for inclusion, excluding those not relevant to the focus, the articles that were not accessible and the articles that provided only theoretical training. Considering the type of articles analyzed and their content, it was not possible to perform a systemic review.

## 3. Results

A total of 97 articles were included in the initial search. Eleven studies were deemed relevant and were considered eligible for full-text reading. Among these, ten studies focused on the analysis of training effectiveness. These studies evaluated training in robotic surgery across various specialties, including gynecology, urology, and general surgery. An article in our review assessed the benefits of introducing preoperative and intraoperative checklists and sign out at the end of the procedure in the operating room. Among the studies that analyzed training in robotic surgery, 9 provided a detailed description of the number of participants and training sessions, totaling 220 participants and 2541 training sessions. The primary training methodology examined was virtual reality, assessed in five studies (50%), followed by dry lab training (four studies, 40%). Two studies compared virtual reality with dry lab training. One study examined cadaver-based training, while one compared single-console with double-console training.

The characteristics of the selected studies are summarized in Table 1.

## 4. Discussion

Our analysis shows that all training and simulation techniques help reduce the rate of errors and complications in robotic surgery. With the increasing complexity of the systems used in surgery, the adequate training of the entire team and the development of checklists have become essential criteria for patient safety. To date, we are still far from the complete implementation of the aeronautical model in gynecological robotic surgery. However, the increasing use of checklists, along with more extensive training, are fundamental steps in this direction.

Based on the Air Force’s know-how in creating checklists and their importance, a program was launched in the U.S. in 2008 to standardize high-risk processes in obstetrics. Obstetric situations with a high risk of errors such as postpartum hemorrhage and severe hypertension were evaluated. The task force developed and implemented the checklists. Subsequently, doctors and nurses were interviewed about the perceived effects of the checklists on patient safety. The checklists were evaluated as very useful for the clinical team in providing good care [31].

Nowadays, checklists have widely entered clinical practice and are used by all hospitals. The surgical “timeout” is the most widely recognized checklist in the medical field. The World Health Organization (WHO) preoperative checklist has been shown to reduce surgery-related mortality and surgical site infections [32]. Checklist items include confirmation of the patient’s identity, the surgical site, and questions regarding comorbidities or potential complications. After the implementation of the WHO preoperative checklist, the results showed a reduction in major surgical complications and mortality by about one-third. [33]. Subsequently, a specific checklist for robotics surgery was developed. McCarroll et al. evaluated the rate of readmission before and after introducing specific preoperative and intraoperative checklists for robot-assisted gynecological procedures. The results showed a significant readmission reduction after the checklist introduction (*p* = 0.02). The duration of surgery, on the other hand, was not significantly influenced (*p* = 0.40). This study demonstrates how integrating a specific checklist for robotic-assisted gynecological surgery significantly reduces readmissions without significantly impacting operating room time [30]. All team members’ implementation and support of checklists have improved patient care and can effectively reduce medical errors, standardize care, and improve communication in medicine [34].

Despite the implementation of checklists, errors in surgery continue to occur. As demonstrated by the FDA MAUDE (U.S. Food and Drug Administration—Manufacturer and User Facility Device Experience) database analysis, 280 adverse events in gynecological robotic surgery with the DaVinci system were recorded between January 2006 and December 2012. Among the reported events, 26% resulted in injury, and 8.5% resulted in death. It should be noted that although procedures on the annexes were performed in less than 3% of the cohort, they accounted for 20% of deaths. Operator errors accounted for 21% of injuries, and 14% were attributed to technical system failures. Sixty-five percent were not directly related to the robot used. In four cases, the surgeon looked away from the surgical field without removing their head from the console, and simultaneously moved the instruments inside the patient [35]. This analysis shows that adverse events still occur in robotic surgery. This surgical technique involves interactions between a complex machine and the operator. The FDA’s analysis shows that a significant percentage of errors are not due to the technical expertise of the surgical team but to an incorrect interaction between the operator and the machine. As a result, adequate theoretical and practical training through simulation can positively affect the reduction in adverse events.

Grogan et al. evaluated 489 CRM training participants, of whom 468 completed an end-of-course interview. The results were very positive, with 95% of respondents believing that CRM training would reduce errors in their daily practice and improve several aspects, such as fatigue management, team building, communication, adverse event recognition, team decision making, and performance feedback [11].

In the study by Zattoni et al., two urological teams were subjected to the simulation of 10 conversions during robotic partial nephrectomy operations and 4 h of theoretical lessons but in reverse order. Although the results did not reach statistical significance, they showed that the group that received theoretical training before the simulations had a reduced number of errors and a shorter conversion time to open surgery. However, both groups showed decreases in mistakes and conversion time to open surgery after training [20]. In intraoperative crises, simulations could effectively improve teamwork and facilitate timely conversions to open surgery, as demonstrated by the study by Zattoni et al. [20].

Training in surgical practice is essential, and with increasing technological innovation, more and more new simulation methods have been developed. Currently, we can divide training in robotic surgery into three areas: inanimate and animate simulation (including cadavers), virtual reality (VR) simulators, and dual consoles in robotic training [36].

With the increase in simulation methods, major certification agencies with training programs have started developing courses for robotic surgery. For example, in GESEA Level 2 Robotics, after a test to verify knowledge on instrumentation, endoscopic techniques, and the management of complications, inanimate simulations are performed with the robot to evaluate psychomotor skills. These simulations focus on camera manipulation, hand–eye coordination, and bi-manual coordination [19].

Robotics training using cadavers has been reported to be effective for improving robotic skills but is predominantly used for advanced robotics training due to high costs [21]. Only some inanimate models have been developed and validated for robotic surgical training [22,23,24]. Both VR and dry lab simulation are effective in improving robotic surgical skills, but they are not equivalent. as indicated by Raison et al.‘s work: dry lab training is superior to VR simulation for more advanced training [25,26]. However, like cadaveric laboratories, the dry lab requires access to a robotic system and training tools, which are not always accessible. Virtual reality simulators are the method mainly used for surgeon training. Studies have shown that repetitive practice on a robotic simulator can improve the performance of simulation capabilities [27]. However, determining which simulator exercises are most effective for training the gynecological procedure has yet to be well studied [36]. In the study of Culligan et al., there were no differences in the hysterectomy performance scores in live patients between novice surgeons who completed simulator training and surgeons who did not. This study found that simulator training shortened the learning curve for robotic surgery, with measurable differences in mean operating time and estimated blood loss [28]. One of the latest innovations is dual-console robotic training; this offers a more significant “hands-on” experience with longer console time, more surgical steps performed, and more interaction with the mentoring surgeon than single-console training [29]. The study of Leon et al. showed that this technique provides a viable alternative in robotic training without increasing complications or surgical time.

As demonstrated by the articles in the literature, training and simulation are effective in reducing the number of errors during surgical procedures. However, the main challenges with training remain the high cost and lack of facilities [21,25,26]. The use of an appropriate checklist would optimize console time and reduce operating room time and thus costs. In addition, greater efficiency would allow for better use of the platform and enable surgeons to perform even more procedures.

As far as we know, this is the first review that tried to assess the evolution of training and medical safety based on innovations introduced by aviation.

## 5. Conclusions

Considering what happened in aeronautics, there has been increasing emphasis on greater rigor in the medical field to reduce errors. Innovations created in the field of aviation, such as checklists and simulation, have entered the medical field to prevent human error.

Tools such as checklists, training, and simulation are among the best methods to reduce adverse medical events. However, eliminating these types of errors is utopian. Technological developments will undoubtedly contribute to reducing human errors in the operating room, thanks to the development of increasingly advanced simulation techniques.

In the future, the introduction of artificial intelligence in operating theatres and the use of augmented reality may allow for greater rigor and the systematization of control processes in medicine, particularly in robotic surgery.

Developing dedicated checklists for use by surgical teams, through theoretical and practical training, has become essential in modern medicine. This training makes it possible to significantly reduce operating times and complications, resulting in lower costs.

## Figures and Tables

**Table 1:** Prospective studies on robotic surgery examined.

Author	Title	Focus	Type of Training	No. of Training Session	No. of Participants	Results	Conclusions
F. Zattoni et al. [20]	Development of a surgical safety training program and checklist for conversion during robotic partial nephrectomies	Training	Inanimate simulation	20	-	No. of errors (median of 2 groups): Before training: 8.5 After training: 0.5	Open conversion simulations might improve teamwork and facilitate timely conversions to open surgery
R. Bertolo et al. [21]	Single session of robotic human cadaver training: the immediate impact on urology residents in a teaching hospital	Training	Cadaveric training	27	27	Post-training improvement: Port placement and docking (*p* = 0.009) EndoWrist manipulation (*p* = 0.002) Fourth arm integration (*p* = 0.002) Needle control and driving (*p* < 0.001)	Human cadaver robotic training allowed for immediate improvement in robotic skills
P. Ramos et al. [22]	Face, content, construct, and concurrent validity of dry laboratory exercises for robotic training using a global assessment tool	Training	Dry lab training	-	26	Dry lab training rating (0–10): Realism: 8 Utility: resident (9), fellow (7), expert (4)	These results demonstrate the usefulness of dry lab training
C.Y. Ro et al. [23]	A novel drill set for the enhancement and assessment of robotic surgical performance	Training	VR simulator	147	21	Post-training improvement: Precision beads (*p* < 0.001) Simple rope pass (*p* = 0.003) Russian roulette (*p* < 0.001) Minefield (*p* = 0.110) Suturing (*p* = 0.080)	The robotic learning curve for novices reflected an improvement in scores (*p* < 0.05)
G. Dulan et al. [24]	Proficiency-based training for robotic surgery: construct validity, workload, and expert levels for nine inanimate exercises	Training	Dry lab training	432	12	Experts vs. novices (composite score): 932 ± 67 vs. 618 ± 111 (*p* < 0.001)	Using objective performance metrics, all exercises demonstrated construct validity
N. Raison et al. [25]	Virtually competent: a comparative analysis of virtual reality and dry-lab robotic simulation training	Training	VR simulator vs. dry lab training	129	43	Improvement in technical proficiency dry lab vs. VR session (mean GEARS score): 1st: 10.5 vs. 11.8 (*p* = 0.060) 2nd: 12.0 vs. 12.6 (*p* = 0.370) 3rd: 16.1 vs. 14.3 (*p* = 0.030)	Dry lab training showed significantly greater improvements than VR simulation after 3 training sessions
L.K. Newcomb et al. [26]	Correlation of virtual reality simulation and dry lab robotic technical skills	Training	VR simulator vs. Dry lab training	300	30	Spearman’s correlation coefficients between corresponding VR and dry lab drills (overall score): 0.87 (*p* < 0.010)	VR drills were found to have a statistically significant correlation with the corresponding dry lab drills
S.S. Sheth, et al. [27]	Virtual reality robotic surgical simulation: an analysis of gynecology trainees	Training	VR simulator	1360	34	Post-training improvement: Matchboard (*p* < 0.001) Ring and rail (*p* < 0.001) Suture sponge (*p* < 0.001) Energy switching (*p* < 0.001)	Virtual reality robotic simulation enhances skills at all training levels
P. Culligan et al. [28]	Predictive validity of a training protocol using a robotic surgery simulator	Training	VR simulator	-	19	Surgery results (surgeons after training vs. control surgeons): Mean operative times for hysterectomy: 21.7 vs. 30.9 (*p* < 0.001) Mean estimated blood loss: 25.4 vs. 31.25 (*p* < 0.001) Mean GOALS scores: 34.7 vs. 31.1 (*p* = 0.070)	Completing the simulator training seems to have reduced the learning curve for beginner robotic surgeons
M.G. Leon, et al. [29]	Impact of robotic single and dual console systems in the training of minimally invasive gynecology surgery (MIGS) fellows	Training	Dual consoles	126	8	Dual console vs. single console training: Console time (*p* < 0.001) Number of steps performed (*p* = 0.009) Surgical takeovers (*p* < 0.001)	Dual console robotic training provides fellows the opportunity for longer console time, a higher number of surgical steps performed, and added interaction with the attending surgeon
M.L. McCarroll et al. [30]	Development and implementation results of an interactive computerized surgical checklist for robotic-assisted gynaecologic surgery	Checklist	-	-	-	Thirty-day readmissions: Pre-checklist: 12 Post-checklist: 5 (*p* = 0.020)	Integrating a specific checklist for gynecologic robotic-assisted surgery resulted in a significant reduction in readmissions

## Data Availability

All data analyzed in this review can be found in the individual articles cited and are available on PubMed. The DOI of the individual articles can be found in the “References” section.
